# Genetics of Perceived Family Interaction From 12 to 17 Years of Age

**DOI:** 10.1007/s10519-019-09960-z

**Published:** 2019-05-24

**Authors:** Karri Silventoinen, Jinni Su, Lea Pulkkinen, Peter Barr, Richard J. Rose, Danielle M. Dick, Jaakko Kaprio

**Affiliations:** 1 Department of Social Research, University of Helsinki, P.O. Box 18, FIN-00014 Helsinki, Finland; 2 Department of Psychology, Virginia Commonwealth University, Richmond, VA USA; 3 Department of Psychology, University of Jyvaskyla, Jyvaskyla, Finland; 4 Department of Psychological and Brain Sciences, Indiana University, Bloomington, IN USA; 5 Department of Human and Molecular Genetics, Virginia Commonwealth University, Richmond, VA USA; 6 College Behavioral and Emotional Health Institute, Virginia Commonwealth University, Richmond, VA USA; 7 Institute for Molecular Medicine (FIMM), University of Helsinki, Helsinki, Finland; 8 Department of Public Health, University of Helsinki, Helsinki, Finland

**Keywords:** Family interaction, Adolescents, Twins, Genetics

## Abstract

We analyzed how the effects of genetic and environmental factors on the perceptions of family interaction change from early to late adolescence. The data were collected by postal surveys on Finnish twins (N = 4808) at 12, 14 and 17 years of age and analyzed using genetic twin modeling. Additive genetic factors explained a modest share of the variation in perceived relational support (a^2^ = 0.30 in boys and 0.18 in girls) and relational tensions (a^2^ = 0.13 and 0.14, respectively) at 12 years of age, with the proportions becoming larger through 17 years of age (a^2^ = 0.53 in boys and 0.49 in girls for relational support; a^2^ = 0.35 in boys and 0.33 in girls for relational tensions). Simultaneously, the role of environment shared by co-twins decreased. These findings suggest that the associations between perceived family interaction and other factors in adulthood should be interpreted with caution, because they partly reflect genetic background, whereas in childhood, they may provide more reliable information on parental characteristics.

**Electronic supplementary material:**

The online version of this article (10.1007/s10519-019-09960-z) contains supplementary material, which is available to authorized users.

## Introduction

Previous studies have shown that the psychosocial family environment has important influences on offspring, including effects on mental health (Yap and Jorm 2015), health behaviors such as physical activity (Beets et al. 2010), and other health-related outcomes such as body mass index (BMI) (Sokol et al. 2017). There is convincing evidence, however, that the psychosocial family environment cannot be considered as a purely environmental factor, since it also reflects the genetic background of the child. A review by Klahr and Burt (2014) demonstrated that the family environment, as reported by children, shows moderate heritability when estimated by using a genetic twin design. The psychosocial family environment has many aspects and can be defined in different ways, but the quality of the parent–child relationship and how offspring perceive their family atmosphere have been regarded as particularly important and are used especially in the previous behavioral genetic studies (Klahr and Burt 2014). Thus, a part of the variation in the family environment as measured by parenting and family interaction as perceived by children is because of inter-individual genetic differences.

There are at least three possible mechanisms explaining this genetic component behind the perceived family environment. First, there is strong evidence based on both twin and molecular genetic studies that the genetic makeup of parents affects their behavior and in turn the family environment (Mileva-Seitz et al. 2016). Because children inherit genes from their parents, a correlation between the genotype of children and their family environment results, which is called the passive gene–environment correlation (Jaffee and Price 2007). Second, children can evoke reactions from their parents due to their personality and other factors that are genetically influenced. For example, a Swedish twin study found that externalizing problems in children, which showed strong heritability, evoked criticism from their mothers (Narusyte et al. 2011). Finally, children may experience the same environmental exposures differently because of differences in personality. Since genetic factors are known to explain individual differences in personality in childhood (Spengler et al. 2012), different perceptions of the family environment can generate genetic differences in the perceived family environment.

The heritability estimates of the perceived family environment may change, however, from childhood to adulthood. Based on the previous literature, adolescence is characterized by decreasing dependence on parents, widening social networks, stronger influence of peers and increased sensation seeking which may lead to increased risk behavior (Ahmed et al. 2015; Kilford et al. 2016). These changes can modify the heritability estimates of the perceived family environment through changing gene–environment correlations. A previous meta-analysis of mainly cross-sectional studies on the heritability of the family environment found that when based on children’s own reports, the influence of environmental factors shared by co-twins decreased and the influence of environmental factors unique to each twin increased during aging; the effect of genetic factors remained roughly stable (Klahr and Burt 2014). This result is consistent with the decreasing influence of parents and increasing influence of peers during adolescence. However, there is also evidence regarding physiological traits such as BMI (Silventoinen et al. 2016) and many psychological traits such as intelligence and mental health indicators (Bergen et al. 2007) that the influence of genetic factors increases during adolescence. A possible reason is that the influence of the passive gene–environment correlation decreases and is partly replaced by the active or evocative gene–environment correlations when children actively shape their environment or evoke reactions from other persons such as their peers (Marceau et al. 2016). This process is demonstrated by multiple studies showing that environmental exposures show moderate heritability (Kendler and Baker 2007). Further, because of increasing independence, personality factors may more strongly shape how adolescents interpret environmental exposures as compared to younger children. Thus, it could also be expected that the influence of genetic factors on how adolescents experience the family environment increases during this developmental period.

We are aware of two longitudinal twin cohorts which have examined the role of genetic factors on the perceived family environment over this transitional period, but these studies have produced somewhat inconsistent results. Both of the studies were based on offspring ratings of the family environment, but the measurement instruments used differed. A US twin study found that the role of genetic factors increased from 11 to 14 years of age in boys and girls using a parent–child relationship questionnaire (McGue et al. 2005). A follow-up study on this same cohort found that the genetic variation increased and the shared environmental variation decreased until 17 years of age; this increasing genetic variation was strongly correlated with genetic variation already present at 12 years of age (Ludeke et al. 2013). In contrast, a UK twin study using three measures of the family environment (household chaos, parental discipline and parental feelings) found no systematic change in the role of genetic and shared environmental factors from nine until 16 years of age (Hannigan et al. 2017a). Further, in this UK study, the genetic correlations between the ages were low. Thus, more longitudinal research is needed to clarify how genetic and environmental influences on family interaction may change across development.

In this study, we analyzed how the genetic architecture of perceived family interaction changes from 12 to 17 years of age by using a longitudinal Finnish twin dataset. Two indicators of family interaction, relational support and relational tensions, were used. We focused on these two dimensions of family interaction because they capture positive and negative aspects of family interaction and have been shown to be predictive of adolescent psychosocial outcomes (Latendresse et al. 2010; Silventoinen et al. 2014). We analyzed both the change in genetic and environmental variation of family interaction and how genetic and environmental factors explained the stability of the perception of family interaction from early to late adolescence.

## Data and methods

The study cohort was derived from the longitudinal FinnTwin12 study covering all Finnish twins born in 1983–1987 (Kaprio et al. 2002). The names and postal addresses of the twins and their parents were received from the Finnish population registry by identifying families with two children born to the same mother on the same day (3136 twin families, supplementary Fig. 1). In response to an invitation to take part in the study, 86% of families indicated their willingness and returned a questionnaire (on the birth, childhood and early school years of the twins) and consent form. After return of the questionnaire, further baseline questionnaires were sent to the parents and twins. The baseline twin questionnaire was sent to both co-twins individually in the autumn of the year they reached the age of 11–12 years (mean age: 11.42 years; range: 11.41–11.43 years). Zygosity was determined based on questionnaire items on the physical similarity and confusability of appearance at school age and, if needed, was supplemented by school photographs and questions for parents. The reliability of this method was validated in a sample of 295 same-sex twin pairs using DNA; zygosity was confirmed among 97% of the pairs, showing good reliability of this method (Jelenkovic et al. 2011). After removing 264 twins with unknown zygosity and 226 twins who were not available or did not respond to the survey, we had 4920 valid responses (49% girls), including 2456 complete twin pairs from which 34% were monozygotic (MZ), 33% same-sex dizygotic (SSDZ) and 33% opposite-sex dizygotic twins (OSDZ). This represents 78% of all Finnish twins in these birth cohorts. Simultaneously when the first questionnaire to twins was sent, a questionnaire was sent to their parents asking for behavioral ratings of the twins and family interactions. This family questionnaire yielded valid responses from 2320 families (74% of all families). A second survey, at the mean age of 14.0 years (range: 13.9–14.9 years; 4523 valid responses; 72% of all twins), was sent to those twins who responded to the first survey and a third survey, at the mean age of 17.6 years (range 17.2–19.5 years; 4041 valid responses; 64% of all twins), was sent to those twins who responded to the second survey.

Items in the family interaction questionnaire asked whether the family was (1) warm, caring; (2) creative, supportive; (3) trusting, understanding; (4) open; (5) strict; (6) unjust; (7) conflicted; and (8) indifferent. A 5-point scale (1 = fits completely, 2 = mainly, 3 = somewhat, 4 = mainly not, 5 = not at all) was used (Narusk and Pulkkinen 1994). The ratings were re-coded such that for all variables higher values indicate better family interactions. The same questions were used in the questionnaires sent to twins at 12, 14 and 17 years of age as well as to parents when their twin children were 12 years of age. Twins were instructed to fill in the questionnaire independently, but parents were asked to do it together. In most of these families, the twins’ mother (60%) or mother and father jointly (35%) completed this family questionnaire. Thus, in nearly all cases, the mother had an important role in the ratings, but the father may have also contributed to these ratings. The parents were asked to consider all children when making the ratings; thus, the parental ratings are the same for both co-twins.

The initial factor analyses showed a 2-factor solution (supplementary Table 1). At all ages and in both boys and girls, the eigenvalues decreased less than one for the third factor; together, the first two factors explained 54–65% of the total variation. When we analyzed the factor loadings of the un-rotated solutions, we found that items 5–8 loaded negatively on the first factor, indicating that these items create another dimension (supplementary Table 2). The factor loadings were largely similar at all ages, for both boys and girls and when using either offspring or parental reports. To create more interpretable results, in the further analyses we conducted two separate 1-factor solutions: for items 1–4 we interpret to indicate relational support and 5–8 to indicate relational tensions. This strategy allowed us to analyze the correlations between these two dimensions of family interaction. When using these two 1-factor solutions, the factors explained 55–73% of the variation; item 5 (strict) did not fit well on either measure reducing the explained variation by 10–14%, and was thus excluded from further analyses (supplementary Table 3).

We then calculated the factor scores of relational support and relational tensions separately by using the 1-factor solutions such as also in the previous studies using this questionnaire (Latendresse et al. 2010; Silventoinen et al. 2014). The Cronbach α-values varied between 0.71 and 0.87 for relational support and between 0.57 and 0.69 for relational tensions, suggesting good internal consistency of these measures (supplementary Table 3). The systematically lower Cronbach α-values for relational tensions was affected by a smaller number of items than was used for relational support because of the removal of one item (strict). In the descriptive analyses, we used sum variables to show how the perceived family interaction changed from 12 to 17 years of age. The scores varied from 4 to 20 for relational support and 3–15 for relational tensions. However, to make the results comparable, we transformed both to vary between 0 and 100 where higher values indicate better support and less tensions. All twins having missing for any item were removed from the analyses of relational support (items 1–4) and relational tensions (items 6–8). The number of valid measures in twins decreased from 4799 for relational tensions and 4808 for relational support at 12 years of age to 3997 and 3988, respectively, at 17 years of age (supplementary Fig. 1). The number of complete twin pairs having these measures at each age by sex and zygosity are available in supplementary Table 4.

**Fig. 1:**
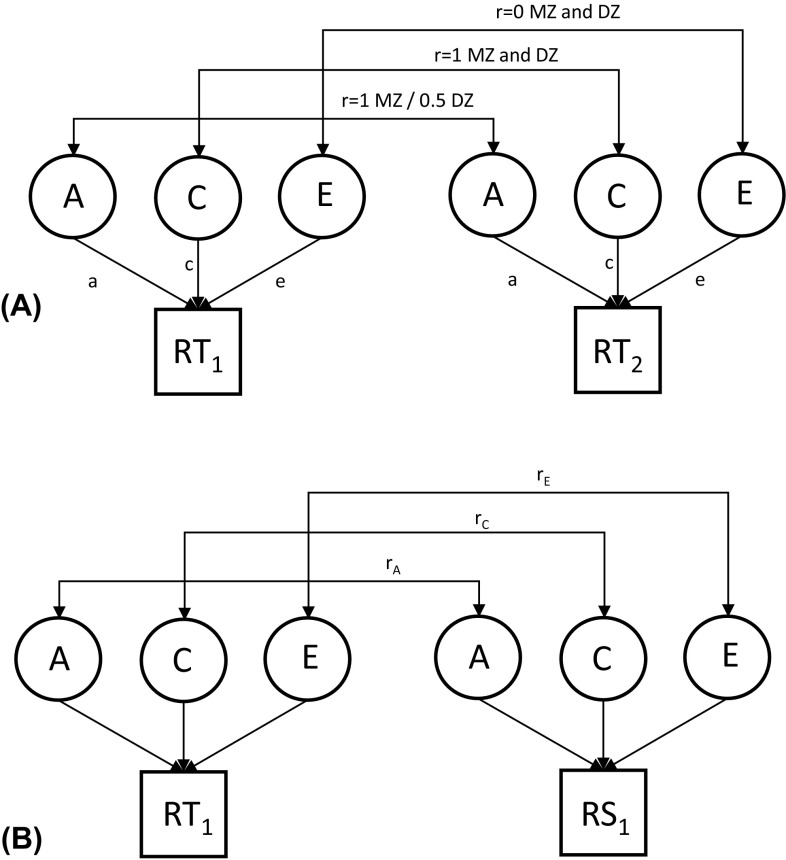
Analytical models: **a** Univariate additive genetic (A), shared environment (C) and unique environment (E) model for relational tensions (RT_1_ for first and RT_2_ for second twin); **b** Bivariate Cholesky decomposition (presented for one twin only) of additive genetic correlation (r_A_), shared environmental correlation (r_C_) and unique environmental correlation (r_E_) between RT and relational support (RS)

The data were analyzed using genetic twin modeling based on the comparisons of similarity between MZ and dizygotic (DZ) twins. MZ twins have virtually the same gene sequence whereas DZ twins share, on average, 50% of their genetic variation (Posthuma et al. 2003). Univariate models were first used to decompose the trait variation in perceived family interactions into three variance components: additive genetic factors (A) including the effects of all loci on the trait (correlation 1.0 within MZ and 0.5 within DZ co-twins), shared environment (C) including the effects of all environmental factors making co-twins similar (correlation 1.0 within both MZ and DZ co-twins) and unique environment (E) including the effects of all environmental factors making co-twins different (correlation 0 both within MZ and DZ co-twins), along with measurement error (Fig. 1a). We found statistically significant correlations between age and relational support at 14 years of age (r = − 0.05; *p* = 0.021), whereas the correlation was marginally significant for relational support at 12 years (*p* = 0.078) and relational tensions at 14 years of age (*p* = 0.054). However, to make the results systematic, we adjusted the results for exact age at all measurements because otherwise the age effect would have been modeled as a part of shared environmental variation. DZ correlations were systematically higher than half of the MZ correlations, suggesting the presence of shared environmental factors (supplementary Table 4). Thus, we used an additive genetic/shared environmental/unique environmental (ACE) model in the analyses. The model fit statistics of the genetic twin models are presented in supplementary Table 5. The assumptions of genetic twin models applied well, as seen in the good fit of the ACE models as compared with the saturated models; the model fit difference was only statistically significant for relational tensions at 17 years of age (*p* = 0.013), but this can also be due to multiple testing since it is larger than the Bonferroni corrected *p* value (*p* = 0.008 for 6 tests). We only found statistically significant sex-specific genetic effects for relational support (*p* = 0.046) and tensions (*p* = 0.009) at 12 years of age, but the OSDZ correlations were also somewhat lower than the SSDZ correlations at the other ages, supporting the sex-specific genetic effect (supplementary Table 4). Thus, to have the internally consistent results, we allowed sex-specific genetic effects at all ages. Differences between boys and girls were highly statistically significant except for relational tensions at 12 years of age. Thus, we stratified all further analyses by sex.

Following the univariate models, we decomposed the covariation between the two dimensions of family interaction at same ages as well as for each measure between different ages by using bivariate Cholesky decomposition (Fig. 1b). This method makes no assumptions about the underlying genetic architecture but decomposes the variance and co-variance in the data into a series of uncorrelated genetic and environmental factors. Using this method, we calculated genetic and environmental correlations between relational tensions and relational support at each age (cross-trait correlations) as well as between these measures at different ages (cross-age correlations). Further, we calculated how much shared genetic and environmental factors explain the total correlations.

The genetic twin models were fitted using the OpenMx package, version 3.0.2, of R statistical software (Neale et al. 2016). The maximum likelihood estimation was used to estimate the parameters and their 95% confidence intervals (CI). For descriptive statistics, statistical tests were performed using linear regression models by Stata/SE 13.1 for Windows statistical software (StataCorp, College Station, TX, USA). In the statistical tests, the effect of intra-pair correlations on standard errors (i.e. sampling twin pairs rather than independent individuals) was taken into account by the cluster option in Stata (Williams 2000).

## Results

Table 1 presents the descriptive statistics for family interaction by sex and zygosity. The offspring ratings of relational support declined from 12 to 17 years of age by 8.3 points (95% CI 7.4–9.3) in boys and by 10.8 points (95% CI 9.8–11.7) in girls. At the same time, the standard deviations (SD) of relational support increased in boys and girls. For relational tensions, the results were less systematic: while in girls the ratings decreased by 4.2 points (95% CI 3.2–5.3), in boys the difference was not statistically significant (*p* = 0.80). Some decrease in SD of relational tensions were seen in boys and girls. Parents reported less relational tensions [7.8 points (95% CI 7.0–8.7) in boys and 5.3 points (95% CI 4.4–6.2) in girls] but also reported relational support to be lower [2.0 points (95% CI 1.2–2.7) in boys and 3.0 points (95% CI 2.2–3.8) in girls] than their twin children reported at 12 years of age. MZ twins experienced slightly better relational support and less relational tensions than DZ twins. The differences were statistically significant (0.71–1.3 points; *p*-values 0.002–0.039 when adjusted for sex) except for relational tensions at 12 years of age (*p* = 0.065). Girls reported better relational support (1.7 points 95% CI 0.94–2.52) and less relational tensions (2.5 points 95% CI 1.6–3.5) at 12 years of age than boys. However, this difference disappeared at 14 years of age, and at 17 years of age, girls reported less relational support (1.3 points 95% CI 0.2–2.4) and more relational tensions (2.1 points 95% CI 1.1–3.1) than boys. Only minor differences were found in SD between MZ and DZ twins. In the comparisons of SSDZ and OSDZ twins, both means and SDs were very similar (data not shown but are available on request). The parental ratings of family interaction when their offspring were 12 years of age correlated modestly with the offspring ratings at the same age [r = 0.32 (95% CI 0.29–0.34) for relational support and r = 0.24 (95% CI 0.22–0.27) for relational tensions]. These correlations somewhat decreased when using offspring ratings at 14 [r = 0.27 (95% CI 0.25–0.30) and r = 0.20 (95% CI 0.18–0.23), respectively] and 17 years of age [r = 0.20 (95% CI 0.17–0.23) and r = 0.17 (95% CI 0.13–0.20), respectively], but the decrease was not substantial.

**Table 1:** Means and standard deviations (SD) of family interaction by age, sex and zygosity

	Boys						Girls					
	All		MZ twins		DZ twins		All		MZ twins		DZ twins	
	Mean	SD	Mean	SD	Mean	SD	Mean	SD	Mean	SD	Mean	SD
Relational support^a^												
12 years of age	84	14	85	13	84	14	86	14	87	14	85	14
14 years of age	80	15	80	15	79	15	79	18	80	17	78	18
17 years of age	76	17	77	17	76	17	75	19	77	19	74	19
Parental report at age 12	82	13	82	12	82	13	83	13	84	12	82	13
Relational tensions^a^												
12 years of age	81	18	82	17	81	18	84	16	85	16	83	16
14 years of age	79	17	81	17	79	17	80	16	82	16	79	16
17 years of age	82	16	83	15	81	16	80	16	81	15	79	16
Parental report at age 12	89	12	89	11	89	12	89	12	90	12	89	12

^a^The scales are transformed to range between 0 and 100

Table 2 presents the results of univariate modeling (Fig. 1a). We allowed the means to differ between MZ and DZ twins in the genetic twin models because of the abovementioned zygosity mean differences. In relational support, additive genetic, shared environmental and unique environmental factors explained roughly equal shares of the variation at 12 years of age in boys, whereas in girls, shared environmental factors were more important and explained nearly half of the variation. The role of genetic factors became more important over time, explaining around half of the variation of relational support at 17 years of age in boys and girls. In relational tensions for both boys and girls, the role of genetic factors was less important than shared and unique environmental factors at 12 years of age. However, genetic factors became more important over time and at 17 years of age explained roughly one-third of the variation. There was some overlap in the 95% CIs of parameters, but generally the differences between the ages were statistically highly significant for relational support (Δ-2LL = 63.4, Δd.f. = 12, *p* < 0.00001) and relational tensions (Δ-2LL = 50.4, Δd.f. = 12, *p* < 0.00001).

**Table 2:** Relative proportions of additive genetic, shared environmental and unique environmental factors with 95% confidence intervals (CI) explaining the variation in family interaction from 12 to 17 years of age

	Additive genetic factors		Shared environmental factors		Unique environmental factors	
	a^2^	95% CI	c^2^	95% CI	e^2^	95% CI
Relational support^a^						
Boys						
12 years of age	0.30	0.14, 0.47	0.34	0.19, 0.47	0.36	0.31, 0.42
14 years of age	0.21	0.02, 0.41	0.36	0.19, 0.51	0.43	0.37, 0.50
17 years of age	0.53	0.31, 0.65	0.06	0.00, 0.25	0.41	0.35, 0.48
Girls						
12 years of age	0.18	0.02, 0.34	0.46	0.31, 0.59	0.36	0.32, 0.42
14 years of age	0.29	0.13, 0.47	0.38	0.21, 0.51	0.33	0.29, 0.39
17 years of age	0.49	0.29, 0.68	0.16	0.00, 0.33	0.35	0.30, 0.41
Relational tensions^a^						
Boys						
12 years of age	0.13	0.01, 0.32	0.39	0.23, 0.50	0.48	0.41, 0.55
14 years of age	0.20	0.01, 0.43	0.27	0.08, 0.44	0.52	0.45, 0.61
17 years of age	0.35	0.20, 0.48	0.05	0.00, 0.18	0.60	0.52, 0.68
Girls						
12 years of age	0.14	0.01, 0.28	0.53	0.40, 0.64	0.33	0.29, 0.38
14 years of age	0.26	0.07, 0.46	0.33	0.15, 0.49	0.41	0.35, 0.47
17 years of age	0.33	0.05, 0.53	0.23	0.06, 0.46	0.43	0.37, 0.50

^a^Factor scores based on two 1-factor models

Finally, we analyzed the correlations between the offspring ratings of relational support and relational tensions (cross-trait correlations) as well as how these dimensions of family interaction correlate between ages (cross-age correlations) (Table 3). As expected, the cross-age correlations were highest between the closest ages (12 vs. 14 years and 14 vs. 17 years) but remained moderate (r ≤ 0.42). Generally, the correlations were similar in boys and girls. The correlations between relational support and relational tensions were lowest at 12 years of age, increasing until 17 years of age to 0.54 in boys and 0.65 in girls. When we decomposed these correlations into genetic and environmental correlations using Cholesky decomposition (Fig. 1b), the highest correlations were generally found for shared environmental factors. Most of these exceeded 0.60, and some exceeded 0.90. Shared environmental factors also explained an important share of the cross-trait and cross-age correlations. Additive genetic correlations were mostly significantly positive and explained a part of these trait correlations. However, most of them were lower than shared environmental correlations. One of the additive genetic correlations was negative leading to the negative proportion of explained variation, but it was not statistically significant. Generally, unique environmental cross-age correlations were small, and around half of them were not statistically significant. For cross-trait correlations, unique environmental correlations were larger than those for cross-age correlations, but they were still smaller than shared environmental and genetic cross-trait correlations.

**Table 3:** Trait correlations of family interaction from 12 to 17 years of age and correlations between additive genetic, shared environmental and unique environmental variance components explaining these trait correlations with 95% confidence intervals (CI)

		Trait correlation		Additive genetic correlation			Shared environmental correlation			Unique environmental correlation		
Trait 1	Trait 2	r	95% CI	r_A_	95% CI	% of the trait correlation explained	r_C_	95% CI	% of the trait correlation explained	r_E_	95% CI	% of the trait correlation explained
Cross-age correlations												
Boys												
Support12	Support14	0.42	0.38, 0.45	0.14	− 0.61, 0.53	9	0.96	0.71, 1.00	77	0.15	0.05, 0.26	14
Support12	Support17	0.25	0.21, 0.29	0.06	− 0.25, 0.30	9	1.00	0.64, 1.00	85	0.04	− 0.06, 0.15	6
Support14	Support17	0.41	0.37, 0.44	0.68	0.38, 1.00	56	0.82	0.23, 1.00	41	0.03	− 0.09, 0.14	3
Tensions12	Tensions14	0.33	0.29, 0.36	0.64	0.22, 1.00	40	0.63	0.36, 1.00	57	0.02	− 0.07, 0.12	3
Tensions12	Tensions17	0.17	0.13, 0.22	− 0.30	− 1.00, 0.07	− 24	0.52	0.30, 1.00	81	0.14	0.05, 0.22	43
Tensions14	Tensions17	0.32	0.28, 0.36	0.30	− 1.00, 1.00	16	0.82	0.49, 1.00	58	0.15	0.04, 0.25	26
Girls												
Support12	Support14	0.39	0.35, 0.43	0.43	0.00, 0.91	29	0.67	0.46, 0.92	66	0.06	− 0.05, 0.16	5
Support12	Support17	0.28	0.24, 0.32	0.09	− 0.45, 0.94	9	0.78	0.38, 1.00	78	0.10	− 0.01, 0.21	13
Support14	Support17	0.42	0.38, 0.45	0.34	0.09, 0.59	34	0.97	0.52, 1.00	52	0.17	0.06, 0.26	14
Tensions12	Tensions14	0.31	0.27, 0.34	0.43	0.00, 1.00	28	0.54	0.26, 0.79	74	− 0.02	− 0.13, 0.09	− 2
Tensions12	Tensions17	0.19	0.15, 0.23	0.41	0.14, 0.92	38	0.35	0.17, 0.65	75	− 0.06	− 0.16, 0.03	− 13
Tensions14	Tensions17	0.32	0.28, 0.36	0.14	− 0.77, 0.44	13	0.71	0.45, 1.00	64	0.17	0.07, 0.28	23
Cross-trait correlations												
Boys												
Support12	Tensions12	0.41	0.38, 0.45	0.64	0.29, 0.96	37	0.62	0.46, 0.82	52	0.12	0.02, 0.21	11
Support14	Tensions14	0.46	0.42, 0.49	0.59	0.10, 1.00	28	0.78	0.53, 1.00	51	0.20	0.10, 0.30	21
Support17	Tensions17	0.54	0.50, 0.57	1.00	0.71, 1.00	62	0.38	− 0.42, 0.96	8	0.31	0.22, 0.40	30
Girls												
Support12	Tensions12	0.44	0.41, 0.47	0.98	0.70, 1.00	45	0.51	0.38, 0.62	53	0.02	− 0.07, 0.11	2
Support14	Tensions14	0.61	0.58, 0.63	0.79	0.49, 1.00	35	0.76	0.59, 0.97	44	0.35	0.26, 0.44	21
Support17	Tensions17	0.65	0.63, 0.68	0.90	0.68, 1.00	51	0.90	0.56, 1.00	27	0.36	0.28, 0.45	22

## Discussion

In this study based on a longitudinal Finnish twin dataset, we found that genetic factors explained an increasing share of the variation of perceived family interaction from 12 to 17 years of age measured as relational support and relational tensions. The results on the influence of genetic factors on family interaction are not surprising, and furthermore, a genetic component has been found not only for family environment (Klahr and Burt 2014) but also for life events and social support (Kendler and Baker 2007), which have been traditionally regarded as part of one’s environment. We found moderate genetic correlations between the ratings of family interaction from 12 to 17 years of age. The US study based on the Minnesota Twin Cohort also found increasing genetic variation and genetic correlations from 11 to 17 years of age when they analyzed parent–child relationships (Ludeke et al. 2013; McGue et al. 2005). In this US cohort, both heritability estimates and genetic correlations were, however, higher than what we found for parental tensions, which was due to a lesser role of shared environmental factors. The UK twin study using the measures of household chaos, parental discipline and parental feelings found that genetic factors explained a smaller and shared environmental factors explained a larger proportion of variation than in our study (Hannigan et al. 2017a). This UK study differed from both our results and the results of the US study because no systematic increase in the role of genetic factors was observed and the genetic correlations were generally of small magnitude. Our results also differ from the results of previous meta-analysis of mainly cross-sectional studies finding that the effect of genetic factors remained roughly stable during adolescence (Klahr and Burt 2014). However, it is noteworthy that different measures of family environment were used in these three longitudinal cohorts and several different measures of family environment were used in the meta-analysis of cross-sectional studies. It is uncertain whether these differences are because of the differences in the measures of family environment, contextual differences or whether they reflect other differences between the cohorts.

In addition to genetic factors, shared environmental factors also explained an important share of the variation in family interaction, especially at 12 years of age. This suggests that especially among children, the ratings of family interaction reflect the characteristics of parenting and less so the children’s own perceptions of parenting characteristics. This conclusion is further supported by our results that shared environmental correlations were substantial and explained in general a larger share of cross-age correlations than genetic factors. Our results on the importance of the shared environment in the stability over ages somewhat differ from a previous review on genetic studies of behavioral problems finding that the stability is mainly influenced by genetic factors (Hannigan et al. 2017b). Naturally, family interaction is not independent of shared genes, but is influenced by parenting which also has a genetic background (Mileva-Seitz et al. 2016). We found modest correlations between parental and offspring ratings of family interaction, which also includes the passive gene–environment correlation. Shared environmental effects become smaller from 12 to 17 years of age when genetic factors become more important. This result is similar to that found for many psychological traits, such as intelligence and mental health indicators (Bergen et al. 2007), and even for BMI (Silventoinen et al. 2016). Since adolescence is characterized by decreasing dependence on parents and increasing influence of peers (Ahmed et al. 2015; Kilford et al. 2016), we could speculate that this increasing genetic effect reflects the role of an active gene–environment correlation when children actively shape their environment partly based on their genotype. Increasing independence may also lead children to interpret family interactions more through their own characteristics, such as personality, which are affected by genetic factors (Spengler et al. 2012).

Unique environmental factors also explained a share of variation of family interaction, and at some ages, they were more important than genetic or shared environmental factors. However, in contrast to genetic and shared environmental factors, the unique environmental correlations were small in size especially when considering the measurements between ages. Moderate unique environmental correlations were, however, found between the two dimensions of family interaction measured at the same ages. This suggests that unique experiences related to family interaction are time-specific. This small effect of unique environmental factors on the stability of family interaction characteristics is supported by a previous twin study which found very low unique environmental correlations for parent–child interaction, even when measured on consecutive days (Burt et al. 2015). This may suggest that parents do not systematically favor or discriminate against the same child over time, and thus unique environmental factors reflect transient effects of probably very minor environmental influences, changes in mood or possible correlated measurement errors since relational support and relational tensions were measured using the same questionnaire.

In addition to the genetic architecture of the family interaction, our data allowed us to study how the perceived family interaction changes from early to late adolescence as well as differ between parents and children. Previous studies have suggested that parent–child relationships deteriorate from childhood to adolescence (Hadiwijaya et al. 2017; Kim et al. 2001; Loeber et al. 2000). Our results only partly supported this because we found deterioration in relational support from 12 to 17 years of age, but when studying relational tensions, the only difference was less relational tensions at 12 years of age in girls. However, the age pattern may depend on how parenting and family interaction have been assessed. In this study, the family-interaction questionnaire was used (Narusk and Pulkkinen 1994), and based on this questionnaire, the two dimensions were calculated such as also in the previous studies using this same measurement instrument (Latendresse et al. 2010; Silventoinen et al. 2014). The decision to use two dimensions was initially based on the results of the explorative factor analyses systematically suggesting the two-factor solution at all ages, in boys and girls as well as in parents and their offspring but was further supported by our empirical results. The decreasing scores of relational support may be related to the increasing activities of children outside home when some children think that they do not anymore get, or even need, support from their parents. This may also explain why the variation of relational support increases at the same time. On the other hand, the increasing independence does not increase tensions in the family and seems actually to level off differences between children as indicated by the decreasing variation of relational tensions. The presence of two dimensions is also supported by comparisons between parental and offspring ratings when the offspring were at the age of 12. Parents rated less relational tensions but lower relational support than did offspring. This may reflect parents’ self-critical attitudes toward family interaction while having more positive attitudes toward their children, rather than vice versa. Thus, our results suggest that relational-support and relational-tensions are correlated but still partly independent dimensions of family-interaction.

Our study has both strengths and weaknesses. The main strength is the longitudinal measures of family interaction using the same questions at three ages covering the critical phase of life from early to late adolescence in a large set of twins, allowing us to study the role of genetic and environmental factors over time. Using the Finnish population register, we were able to identify all twins in the selected birth cohorts. Though the response rates in our twin surveys were very high (88–95%), the repeated surveys decreased retention over time, such that by 17 years of age 64% of all pairs were still in the study. Nonetheless, our study cohort can be regarded as representative. Further, the parents of twins also independently rated family interaction using the same questions as their offspring allowing us to study the associations between the ratings of parents and offspring. The main limitation of our data is that family interaction was assessed with a limited number of questions. A larger number and greater breadth of questions would have probably reduced the measurement error.

In conclusion, genetic factors play an important role in perceived family interaction in late adolescence, whereas at younger ages shared environmental factors are more important. Results using self-reported family interaction in late adolescence and adulthood should be regarded critically because they also reflect genetic background.

## Electronic Supplementary Material

Below is the link to the electronic supplementary material.

Supplementary material 1 (DOCX 52 kb)

## References

[CR1] Ahmed SP, Bittencourt-Hewitt A, Sebastian CL (2015). Neurocognitive bases of emotion regulation development in adolescence. Dev Cogn Neurosci.

[CR2] Beets MW, Cardinal BJ, Alderman BL (2010). Parental social support and the physical activity-related behaviors of youth: a review. Health Educ Behav.

[CR3] Bergen SE, Gardner CO, Kendler KS (2007). Age-related changes in heritability of behavioral phenotypes over adolescence and young adulthood: a meta-analysis. Twin Res Hum Genet.

[CR4] Burt SA, Klahr AM, Klump KL (2015). Do non-shared environmental influences persist over time? An examination of days and minutes. Behav Genet.

[CR5] Hadiwijaya H, Klimstra TA, Vermunt JK, Branje SJT, Meeus WHJ (2017). On the development of harmony, turbulence, and independence in parent-adolescent relationships: a five-wave longitudinal study. J Youth Adolesc.

[CR6] Hannigan LJ, McAdams TA, Plomin R, Eley TC (2017). Parent- and child-driven effects during the transition to adolescence: a longitudinal, genetic analysis of the home environment. Dev Sci.

[CR7] Hannigan LJ, Walaker N, Waszczuk MA, McAdams TA, Eley TC (2017). Aetiological influences on stability and change in emotional and behavioural problems across development: a systematic review. Psychopathol Rev.

[CR8] Jaffee SR, Price TS (2007). Gene-environment correlations: a review of the evidence and implications for prevention of mental illness. Mol Psychiatr.

[CR9] Jelenkovic A, Ortega-Alonso A, Rose RJ, Kaprio J, Rebato E, Silventoinen K (2011). Genetic and environmental influences on growth from late childhood to adulthood: a longitudinal study of two Finnish twin cohorts. Am J Hum Biol.

[CR10] Kaprio J, Pulkkinen L, Rose RJ (2002). Genetic and environmental factors in health-related behaviors: studies on Finnish twins and twin families. Twin Res.

[CR11] Kendler KS, Baker JH (2007). Genetic influences on measures of the environment: a systematic review. Psychol Med.

[CR12] Kilford EJ, Garrett E, Blakemore SJ (2016). The development of social cognition in adolescence: an integrated perspective. Neurosci Biobehav Rev.

[CR13] Kim KJ, Conger RD, Lorenz FO, Elder GH (2001). Parent-adolescent reciprocity in negative affect and its relation to early adult social development. Dev Psychol.

[CR14] Klahr AM, Burt SA (2014). Elucidating the etiology of individual differences in parenting: a meta-analysis of behavioral genetic research. Psychol Bull.

[CR15] Latendresse SJ, Rose RJ, Viken RJ, Pulkkinen L, Kaprio J, Dick DM (2010). Examining the etiology of associations between perceived parenting and adolescents’ alcohol use: common genetic and/or environmental liabilities?. J Stud Alcohol Drugs.

[CR16] Loeber R, Drinkwater M, Yin Y, Anderson SJ, Schmidt LC, Crawford A (2000). Stability of family interaction from ages 6 to 18. J Abnorm Child Psychol.

[CR17] Ludeke S, Johnson W, McGue M, Iacono WG (2013). Genetic amplification and the individualization of the parent-child relationship across adolescence. Psychol Med.

[CR18] Marceau K, Knopik VS, Neiderhiser JM, Lichtenstein P, Spotts EL, Ganiban JM, Reiss D (2016). Adolescent age moderates genetic and environmental influences on parent-adolescent positivity and negativity: implications for genotype-environment correlation. Dev Psychopathol.

[CR19] McGue M, Elkins I, Walden B, Iacono WG (2005). Perceptions of the parent-adolescent relationship: a longitudinal investigation. Dev Psychol.

[CR20] Mileva-Seitz VR, Bakermans-Kranenburg MJ, van IJzendoorn MH (2016). Genetic mechanisms of parenting. Horm Behav.

[CR21] Narusk A, Pulkkinen L (1994). Parental relationship and adolescents concepts of their interaction with significant others. Eur J Psychol Edu.

[CR22] Narusyte J, Neiderhiser JM, Andershed AK, D’Onofrio BM, Reiss D, Spotts E, Ganiban J, Lichtenstein P (2011). Parental criticism and externalizing behavior problems in adolescents: the role of environment and genotype-environment correlation. J Abnorm Psychol.

[CR23] Neale MC, Hunter MD, Pritikin JN, Zahery M, Brick TR, Kirkpatrick RM, Estabrook R, Bates TC, Maes HH, Boker SM (2016). OpenMx 2.0: extended structural equation and statistical modeling. Psychometrika.

[CR24] Posthuma D, Beem AL, de Geus EJ, van Baal GC, von Hjelmborg JB, Iachine I, Boomsma DI (2003). Theory and practice in quantitative genetics. Twin Res.

[CR25] Silventoinen K, Volanen SM, Vuoksimaa E, Rose RJ, Suominen S, Kaprio J (2014). A supportive family environment in childhood enhances the level and heritability of sense of coherence in early adulthood. Soc Psychiatr Psychiatr Epidemiol.

[CR26] Silventoinen K, Huppertz C, van Beijsterveldt CE, Bartels M, Willemsen G, Boomsma DI (2016). The genetic architecture of body mass index from infancy to adulthood modified by parental education. Obesity.

[CR27] Sokol RL, Qin B, Poti JM (2017). Parenting styles and body mass index: a systematic review of prospective studies among children. Obes Rev.

[CR28] Spengler M, Gottschling J, Spinath FM (2012). Personality in childhood—a longitudinal behavior genetic approach. Pers Indiv Diff.

[CR29] Williams R (2000). A note on robust variance estimation for cluster-correlated data. Biometrics.

[CR30] Yap MB, Jorm AF (2015). Parental factors associated with childhood anxiety, depression, and internalizing problems: a systematic review and meta-analysis. J Affect Disord.

