# Correction to: Genetics of Perceived Family Interaction From 12 to 17 Years of Age

**DOI:** 10.1007/s10519-019-09963-w

**Published:** 2019-07-01

**Authors:** Karri Silventoinen, Jinni Su, Lea Pulkkinen, Peter Barr, Richard J. Rose, Danielle M. Dick, Jaakko Kaprio

**Affiliations:** 1 Department of Social Research, University of Helsinki, P.O. Box 18, FIN-00014 Helsinki, Finland; 2 Department of Psychology, Virginia Commonwealth University, Richmond, VA USA; 3 Department of Psychology, University of Jyvaskyla, Jyvaskyla, Finland; 4 Department of Psychological and Brain Sciences, Indiana University, Bloomington, IN USA; 5 Department of Human and Molecular Genetics, Virginia Commonwealth University, Richmond, VA USA; 6 College Behavioral and Emotional Health Institute, Virginia Commonwealth University, Richmond, VA USA; 7 Institute for Molecular Medicine (FIMM), University of Helsinki, Helsinki, Finland; 8 Department of Public Health, University of Helsinki, Helsinki, Finland

## Correction to: Behavior Genetics (2019) 49:366–375 10.1007/s10519-019-09960-z

The article "Genetics of Perceived Family Interaction From 12 to 17 Years of Age", written by Karri Silventoinen, Jinni Su, Lea Pulkkinen, Peter Barr, Richard J. Rose, Danielle M. Dick, Jaakko Kaprio, was originally published electronically on the publisher’s internet portal (currently SpringerLink) on 24 May 2019 without open access.

With the author(s)’ decision to opt for Open Choice the copyright of the article changed on 13 June 2019 to © The Author(s) 2019 and the article is forthwith distributed under the terms of the Creative Commons Attribution 4.0 International License (http://creativecommons.org/licenses/by/4.0/), which permits use, duplication, adaptation, distribution and reproduction in any medium or format, as long as you give appropriate credit to the original author(s) and the source, provide a link to the Creative Commons license and indicate if changes were made.

The original article has been corrected.

