# Supplementary material for: Genetics of Perceived Family Interaction From 12 to 17 Years of Age
Source: Behav Genet. 2019 May 24;49(4):366–75. doi: 10.1007/s10519-019-09960-z (PMC6554250; doi:10.1007/s10519-019-09960-z)
Supplement: Supplementary file 1 — Supplementary material 1 (DOCX 52 kb) [file 10519_2019_9960_MOESM1_ESM.docx]

Removed:

482 twins not available or not responded

Wave 3 (17 years old)

4041 twins (response rate=79%; the proportion of all twins=64%)

3997 relational support

3988 relational tensions

Removed:

397 twins not available or not responded

Wave 2 (14 years old)

4523 twins (response rate=88%; the proportion of all twins=72%)

4384 relational support

4357 relational tensions

Wave 1 (12 years old)

4920 twins (response rate=96%; the proportion of all twins=78%)

4799 relational support

4808 relational tensions

Supplementary figure 1. Flow-diagram: the number of respondents in each questionnaire, response rates, the proportions of the original cohort and the number of valid responses for family interaction measures.

Removed for this analysis:

264 twins of unknown zygosity

Removed:

226 twins not available or not responded

Removed:

253 families not responded

Parental questionnaire

2320 families (response rate=90%; the proportion of all families=74%)

2300 relations support

2307 relational tensions

Parental questionnaire was mailed to twin parents (n=2573 families) and the Wave 1 twin questionnaire to twins (n=5146 twins)

Initial family questionnaire sent to 3136 families (6272 twins) with twins and was returned by 2705 families (5410 twins; 86%)

Supplementary table 1. Eigenvalues and percentages of explained variation in un-rotated factor analyses by sex.

|  | Eigenvalues | | | | % explained of variation | | | |
| --- | --- | --- | --- | --- | --- | --- | --- | --- |
|  | 12 years | 14 years | 17 years | Parents | 12 years | 14 years | 17 years | Parents |
| Boys |  |  |  |  |  |  |  |  |
| Factor 1 | 3.11 | 3.49 | 3.72 | 3.16 | 38.9 | 43.6 | 46.5 | 39.5 |
| Factor 2 | 1.40 | 1.39 | 1.26 | 1.15 | 17.5 | 17.4 | 15.7 | 14.4 |
| Factor 3 | 0.74 | 0.80 | 0.75 | 0.90 | 9.2 | 10.0 | 9.4 | 11.2 |
| Factor 4 | 0.65 | 0.58 | 0.59 | 0.68 | 8.2 | 7.2 | 7.4 | 8.5 |
| Factor 5 | 0.58 | 0.52 | 0.54 | 0.65 | 7.2 | 6.6 | 6.8 | 8.1 |
| Factor 6 | 0.55 | 0.49 | 0.46 | 0.58 | 6.8 | 6.2 | 5.7 | 7.2 |
| Factor 7 | 0.52 | 0.39 | 0.35 | 0.45 | 6.5 | 4.8 | 4.4 | 5.7 |
| Factor 8 | 0.45 | 0.34 | 0.33 | 0.43 | 5.6 | 4.3 | 4.1 | 5.4 |
| Girls |  |  |  |  |  |  |  |  |
| Factor 1 | 3.28 | 3.98 | 4.05 | 3.05 | 41.0 | 49.7 | 50.6 | 38.1 |
| Factor 2 | 1.36 | 1.09 | 1.13 | 1.32 | 17.0 | 13.6 | 14.1 | 16.5 |
| Factor 3 | 0.75 | 0.77 | 0.69 | 0.89 | 9.4 | 9.7 | 8.6 | 11.2 |
| Factor 4 | 0.61 | 0.58 | 0.60 | 0.66 | 7.6 | 7.3 | 7.5 | 8.2 |
| Factor 5 | 0.58 | 0.49 | 0.49 | 0.61 | 7.2 | 6.2 | 6.1 | 7.6 |
| Factor 6 | 0.54 | 0.47 | 0.47 | 0.58 | 6.7 | 5.9 | 5.9 | 7.3 |
| Factor 7 | 0.48 | 0.31 | 0.29 | 0.50 | 6.0 | 3.9 | 3.7 | 6.2 |
| Factor 8 | 0.41 | 0.30 | 0.28 | 0.40 | 5.1 | 3.7 | 3.5 | 5.1 |

Supplementary table 2. Factor loadings when using un-rotated two-factor solution by sex.

|  | 12 years | | 14 years | | 17 years | | Parents | |
| --- | --- | --- | --- | --- | --- | --- | --- | --- |
|  | Factor 1 | Factor 2 | Factor 1 | Factor 2 | Factor 1 | Factor 2 | Factor 1 | Factor 2 |
| Boys |  |  |  |  |  |  |  |  |
| Warm, caring | 0.701 | 0.334 | 0.643 | 0.276 | 0.802 | 0.204 | 0.767 | 0.159 |
| Creative, supportive | 0.665 | 0.412 | 0.736 | 0.374 | 0.797 | 0.269 | 0.706 | 0.229 |
| Trusting, understanding | 0.719 | 0.345 | 0.748 | 0.330 | 0.812 | 0.228 | 0.767 | 0.185 |
| Open | 0.628 | 0.324 | 0.630 | 0.365 | 0.719 | 0.310 | 0.671 | 0.328 |
| Strict discipline | -0.332 | 0.630 | -0.296 | 0.658 | -0.198 | 0.716 | -0.192 | 0.788 |
| Unjust | -0.618 | 0.462 | -0.644 | 0.451 | -0.581 | 0.502 | -0.630 | 0.327 |
| Conflicted | -0.609 | 0.413 | -0.630 | 0.418 | -0.655 | 0.296 | -0.589 | 0.451 |
| Indifferent | -0.631 | 0.339 | -0.607 | 0.296 | -0.629 | 0.253 | -0.505 | 0.045 |
| Girls |  |  |  |  |  |  |  |  |
| Warm, caring | 0.691 | 0.347 | 0.727 | 0.321 | 0.825 | 0.238 | 0.760 | 0.210 |
| Creative, supportive | 0.727 | 0.369 | 0.769 | 0.362 | 0.808 | 0.240 | 0.698 | 0.298 |
| Trusting, understanding | 0.755 | 0.322 | 0.730 | 0.196 | 0.846 | 0.099 | 0.762 | 0.317 |
| Open | 0.671 | 0.330 | 0.596 | 0.279 | 0.737 | 0.222 | 0.644 | 0.378 |
| Strict discipline | -0.355 | 0.617 | -0.399 | 0.716 | -0.303 | 0.836 | -0.194 | 0.608 |
| Unjust | -0.647 | 0.428 | -0.701 | 0.393 | -0.660 | 0.425 | -0.605 | 0.448 |
| Conflicted | -0.584 | 0.450 | -0.670 | 0.281 | -0.644 | 0.241 | -0.580 | 0.438 |
| Indifferent | -0.605 | 0.354 | -0.608 | 0.118 | -0.674 | -0.076 | -0.499 | 0.422 |

Supplementary table 3. Percent of variation explained by one factor of relational support and relational tensions with and without strict discipline item and Cronbach alpha values by sex.

|  | With strict discipline item | | Without strict discipline item | | Cronbach alpha without strict discipline item | |
| --- | --- | --- | --- | --- | --- | --- |
|  | Boys | Girls | Boys | Girls | Boys | Girls |
| Relational support^1^ |  |  |  |  |  |  |
| 12 years | 48.1 | 51.2 | 59.8 | 63.4 | 0.77 | 0.80 |
| 14 years | 55.3 | 59.1 | 68.8 | 72.3 | 0.71 | 0.75 |
| 17 years | 56.4 | 58.8 | 69.9 | 72.6 | 0.85 | 0.87 |
| Parental report | 49.3 | 49.3 | 61.5 | 61.6 | 0.79 | 0.79 |
| Relational tensions^1^ |  |  |  |  |  |  |
| 12 years | 51.6 | 51.5 | 61.4 | 61.0 | 0.68 | 0.68 |
| 14 years | 51.3 | 51.7 | 61.9 | 61.4 | 0.69 | 0.68 |
| 17 years | 51.4 | 50.1 | 61.5 | 60.9 | 0.69 | 0.68 |
| Parental report | 43.6 | 46.2 | 55.2 | 57.9 | 0.57 | 0.61 |

^1^Unrotated 1-factor solution used.

Supplementary table 4. The number of complete twin pairs and within pair correlations with 95% confidence intervals (CI) of family interaction by sex, zygosity and age.

|  | Boys | | | | | | Girls | | | | | | Opposite-sex  DZ twins | | |
| --- | --- | --- | --- | --- | --- | --- | --- | --- | --- | --- | --- | --- | --- | --- | --- |
|  | MZ twins | | | DZ twins | | | MZ twins | | | DZ twins | | |  |  |  |
|  | N | r | 95% CI | N | r | 95% CI | N | r | 95% CI | N | r | 95% CI | N | r | 95% CI |
| Relational support | | | | | | | | | | | | | | | |
| 12 y | 386 | 0.62 | 0.56, 0.68 | 414 | 0.51 | 0.44, 0.58 | 410 | 0.64 | 0.58, 0.70 | 361 | 0.54 | 0.47, 0.61 | 764 | 0.44 | 0.38, 0.50 |
| 14 y | 337 | 0.56 | 0.48, 0.63 | 360 | 0.47 | 0.39, 0.55 | 377 | 0.66 | 0.60, 0.71 | 322 | 0.53 | 0.44, 0.60 | 671 | 0.35 | 0.28, 0.41 |
| 17 y | 301 | 0.60 | 0.52, 0.67 | 321 | 0.33 | 0.23, 0.43 | 366 | 0.64 | 0.58, 0.70 | 314 | 0.42 | 0.32, 0.51 | 620 | 0.27 | 0.20, 0.34 |
| Relational tensions | | | | | | | | | | | | | | | |
| 12 y | 390 | 0.52 | 0.44, 0.59 | 413 | 0.47 | 0.39, 0.54 | 411 | 0.65 | 0.59, 0.71 | 361 | 0.61 | 0.55, 0.67 | 767 | 0.40 | 0.33, 0.45 |
| 14 y | 339 | 0.47 | 0.39, 0.55 | 354 | 0.38 | 0.29, 0.47 | 367 | 0.59 | 0.52, 0.65 | 312 | 0.47 | 0.37, 0.55 | 673 | 0.26 | 0.19, 0.33 |
| 17 y | 307 | 0.35 | 0.24, 0.44 | 319 | 0.30 | 0.19, 0.39 | 363 | 0.52 | 0.45, 0.59 | 309 | 0.45 | 0.36, 0.53 | 613 | 0.23 | 0.15, 0.30 |

Supplementary table 5. Model fit statistics of genetic twin modeling for family interaction.

|  | Saturated model | | Full ACE model^1^ | | No sex-limitation^2^ | | Same parameters for boys and girls^3^ | |
| --- | --- | --- | --- | --- | --- | --- | --- | --- |
|  | -2LL | d.f. | Δ-2LL | P of Δ-2LL | Δ-2LL | P of Δ-2LL | Δ-2LL | P of Δ-2LL |
| Relational support | | | | | | | | |
| 12 years | 12712.41 | 4756 | 12.17 | 0.592653 | 3.99 | 0.045771 | 1.34 | 0.719657 |
| 14 years | 11010.53 | 4310 | 2.44 | 0.999723 | 1.16 | 0.281466 | 35.28 | <0.000001 |
| 17 years | 10273.03 | 3970 | 13.05 | 0.52259 | 0.36 | 0.548506 | 22.53 | <0.000001 |
| Relational tensions | | | | | | | | |
| 12 years | 11015.62 | 4765 | 8.76 | 0.846111 | 6.9 | 0.00862 | 56.07 | <0.000001 |
| 14 years | 10192.61 | 4284 | 15.43 | 0.349406 | 0.41 | 0.52197 | 19.63 | 0.000203 |
| 17 years | 9478.776 | 3961 | 28.402 | 0.012577 | 0.442 | 0.506159 | 16.058 | 0.001103 |

^1^Δ-2LL compared to the saturated model (14 Δd.f.); ^2^Δ-2LL compared to the full ACE model (1 Δd.f.); ^3^Δ-2LL compared to the full ACE model (3 Δd.f.)
